# Thrombosis as a complication of central venous access in pediatric patients with malignancies: a 5-year single-center experience

**DOI:** 10.1186/2052-1839-14-18

**Published:** 2014-10-01

**Authors:** Verena Wiegering, Sophie Schmid, Oliver Andres, Clemens Wirth, Armin Wiegering, Thomas Meyer, Beate Winkler, Paul G Schlegel, Matthias Eyrich

**Affiliations:** Department of Pediatric Hematology/Oncology and Stem Cell Transplantation, University Children’s Hospital, D31, Josef-Schneider-Straße 2, D-97080 Würzburg, Germany; Division of Pediatric Surgery, University Medical Center ZOM, Würzburg, Germany; Division of Pediatric Radiology, University Department of Radiology, Würzburg, Germany

**Keywords:** Pediatric malignancy, Central venous access, Port, Hickman catheter, Thrombosis

## Abstract

**Background:**

Reliable central venous access (CVC) is essential for hematology–oncology patients since frequent puncture of peripheral veins—e.g., for chemotherapy, antibiotic administration, repeated blood sampling, and monitoring—can cause unacceptable pain and psychological trauma, as well as severe side effects in cases of extravasation of chemotherapy drugs. However, CVC lines still carry major risk factors, including thrombosis, infection (e.g., entry site, tunnel, and luminal infections), and catheter dislocation, leakage, or breakage.

**Methods:**

Here we performed a retrospective database analysis to determine the incidence of CVC-associated thrombosis in a single-center cohort of 448 pediatric oncologic patients, and to analyze whether any subgroup of patients was at increased risk and thus might benefit from prophylactic anticoagulation.

**Results:**

Of the 448 patients, 269 consecutive patients received a CVC, and 55 of these 269 patients (20%) also had a thrombosis. Of these 55 patients, 43 had at least one CVC-associated thrombosis (total number of CVC-associated thrombosis: n = 52). Among all patients, the median duration of CVC exposure was 464 days. Regarding exposure time, no significant difference was found between patients with and without CVC-associated thrombosis. Subclavia catheters and advanced tumor stages seem to be the main risk factors for the development of CVC-associated thrombosis, whereas pharmacologic prophylaxis did not seem to have a relevant impact on the rate of thrombosis.

**Conclusions:**

We conclude that pediatric surgeons and oncologists should pay close attention to ensuring optimal and accurate CVC placement, as this appears the most effective tool to minimize CVC-associated complications.

## Background

For hematology-oncology patients, reliable central venous access (CVC) is essential since frequent puncture of peripheral veins—e.g., for chemotherapy, antibiotic administration, repeated blood sampling, and monitoring—can cause unacceptable pain and psychological trauma, as well as severe side effects in cases of extravasation of chemotherapy drugs [1]. In 1973, the percutaneous catheter made of silicone rubber was first introduced for central vein access [2], and totally implantable access ports were introduced in 1982 [3]. Since 1992, the European children’s cancer study group has recommended CVC, especially the port system, as the preferred device for pediatric patients because of its longer life expectancy, easy care, and improved patient acceptance [4, 5]. Today, CVCs are the gold standard in pediatric oncology departments. However, they still carry several associated risks, including thrombosis; infection (entry site, tunnel infection, and luminal infection); and catheter dislocation, leakage, or breakage [4, 6].

Catheter-associated thromboses remain a frequent complication, and there are ongoing scientific discussions about routine administration of prophylactic anticoagulation in pediatric patients with malignancies. On one hand, CVCs may contribute to the risk of thrombophilia. On the other hand, thrombocytopenia-associated risk of hemorrhage may be further aggravated by prophylactic anticoagulation. Cancer itself is a well-known risk factor for thrombosis development through various simultaneous mechanisms involving venous stasis (immobilization and compression), endothelial damage (cancer-associated and from surgery, chemotherapy, and central venous catheters), and blood hypercoagulation (from expression of tissue factors and cancer-associated pro-coagulants, and associated with therapy using hormones, asparaginase, corticosteroids, etc.) [7, 8]. Among adults, the incidence of venous thromboembolism (VTE) is approximately six-fold higher in patients with cancer and up to 10-fold higher in those patients receiving chemotherapy [9]. During therapy, approximately 20% of adult cancer patients will develop VTE, and this occurrence is an independent prognostic factor representing the second leading cause of mortality [7, 10]. However, data in the pediatric cancer population remain sparse and may be different from the adult population, as patient age and therapy protocol intensity are important contributing factors for thrombosis in healthy individuals [11, 12].

There are currently no guidelines regarding antithrombotic prophylaxis in children with malignancy and CVC. Daily instillation of heparin solution into the catheter lumen is commonly performed to prevent clotting of the catheter tip, but no large controlled study has been undertaken to assess the effectiveness of this procedure in children with cancer. The present study aimed to examine the incidence of CVC-associated thrombosis in our study population (n = 448; from January 01, 2008 until December 31, 2012). We further analyzed whether epidemiologic characteristics (such as tumor entity and patient age and gender) and treatment-related characteristics (such as chemotherapy drug, duration, and position) influenced the frequency of thrombosis. Finally, we investigated whether any specific subgroup appeared to have an increased risk of central venous access thrombosis, and thus might particularly benefit from prophylactic anticoagulation.

## Methods

### Patients and data collection

**Figure 1:**
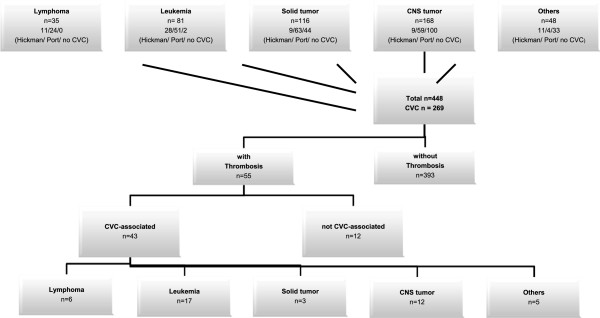
**Flow chart of study populations.**

We performed a systematic search of our electronic patient database for children who were newly diagnosed with malignancy between January 01, 2008 and December 31, 2012. Our research has adhered to the STROBE guidelines for observational studies. Within this population, we further searched for patients who also had central venous access, and suffered from thrombosis or vascular complications unrelated to admission or discharge diagnosis. As in the German health system every diagnosis is encoded according the ICD-10 catalog we searched for I80-82. These ICD10-codes include all kind of thrombosis of the venous system. We performed chart review of all patients, who were identified by this search. Patients were excluded if their reported thrombosis or venous vascular complications were not related to the central venous access (Figure 1). CVC-associated thrombosis was defined as a thrombus at the catheter side which radiologic evidence (by ultrasound, echocardiography or X-ray examination). The clinical evidence of catheter obstruction was facultative, but was present in over 95% of patients.

In accordance with institutional guidelines, informed consent for chart review for retrospective studies was obtained from all patients. Two reviewers (the two first authors) independently extracted epidemiological data (e.g., gender, age, underlying disease, family history, therapy and medication in the preceding 20 days, and catheter details), clinical presentation at the time of thrombosis and during hospital stay, and laboratory findings (e.g., blood gas analysis, full blood count, and coagulation parameters). We could obtain data of all identified patients, so that we did not have to exclude patient for incomplete or missing data. The study was approved by the ethics committee of the University Hospital Würzburg (study #133/04).

### Surgical methods

Following routine protocol, all oncologic patients with an indication for CVC implantation received a port-a-cath. A Hickman catheter was used only in patients undergoing more intensive therapy or with an indication for stem cell transplantation. All CVC implantations were performed under general anesthesia in the Division of Pediatric Surgery. After disinfection, a 3- to 4-cm skin incision was made in the sulcus deltoideus. Subcutaneous tissue was dissected, and the cephalic vein was prepared. If implantation in the cephalic vein was not possible, the vena jugularis externa or interna was prepared instead. Following venotomy, the catheter was inserted and the position checked by intraoperative chest radiography. The catheter was fixed when the tip was located between the junction of the superior caval vein and the right atrium. Catheters were revised immediately in every single patient if the location was not between the junction of the superior caval vein and the right atrium as it is known to be the most evident risk factor for the development of CVC-associated thrombosis. Postoperatively, the catheter tip location was re-checked by chest radiography, blood regurgitation was confirmed, and then the catheter was put into use. The catheter was kept filled with 3 to 5 mL of heparinized saline solution (100 U/mL) after each completion of blood sampling or drug injection. For port systems, port needles were changed under sterile conditions every 14 days.

### Statistical analysis

The data were analyzed using JMP® (version 5.0, software SAS, Camarillo, USA). The analyzed parameters are expressed as mean ± standard error of the mean (SEM) unless otherwise indicated. After data abstraction, the Student’s *t*-test was used for comparative analysis of continuous variables. For statistical analysis, influences of age and gender were ruled out using the exact U test, Fisher’s test, and Puri and Sen’s two-way ranked ANOVA. In all statistical applications, *P* < 0.05 was considered significant.

## Results

Over the 5-year study period, we identified 448 patients with newly diagnosed malignancy (Figure 1). Of these patients, 269 received a central venous access. Prior to CVC implantation, patients were screened for APC resistance, protein S and protein C deficiency, and dysfibrinogenemia. In case of positive findings (one APC resistance, one protein S deficiency, and one protein C deficiency) prophylactic anticoagulation with LMWH was performed during the whole period of CVC-placement, if platelet count was above 40/nL.

**Figure 2:**
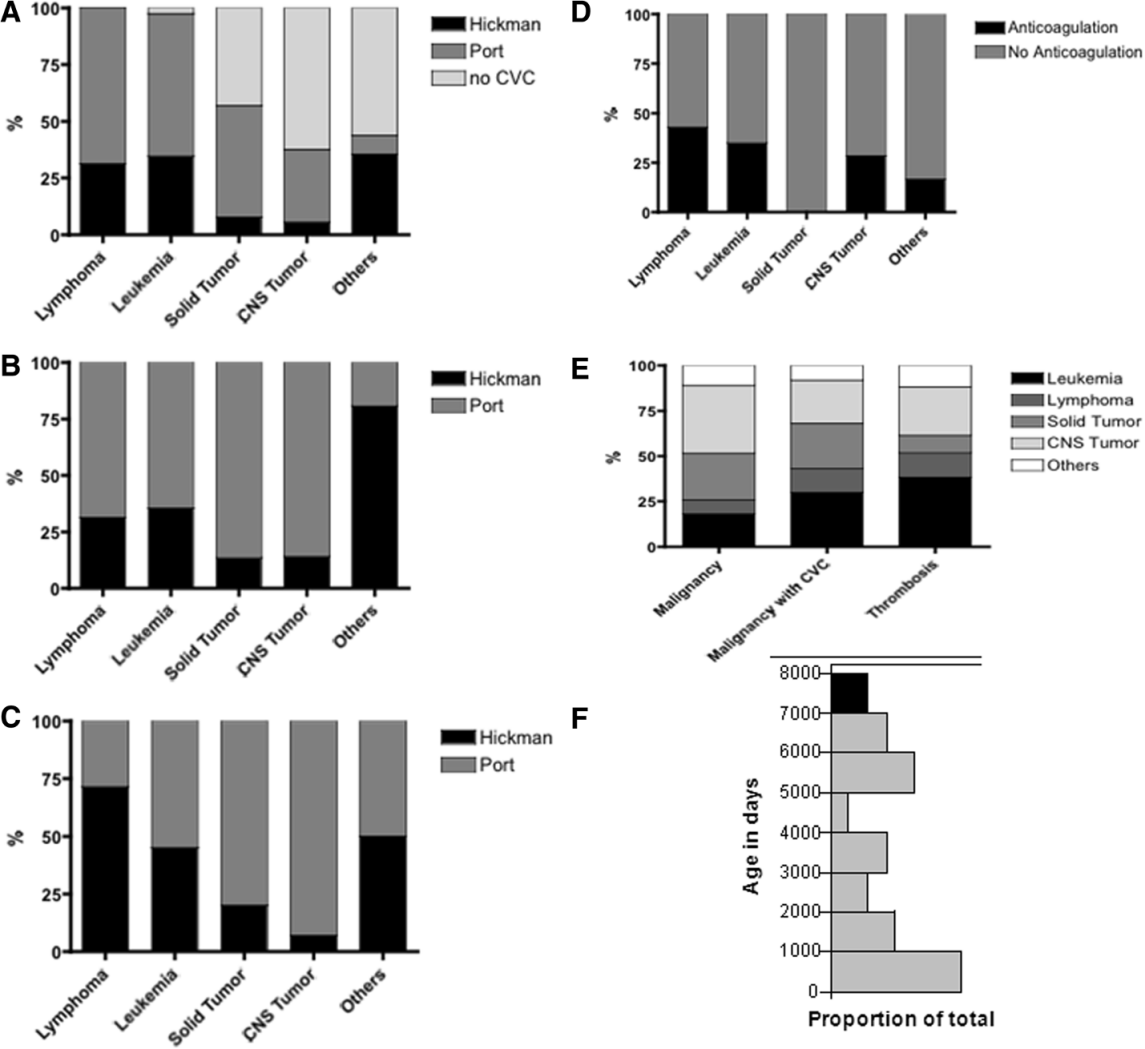
**Thrombosis and CVC distributions. (A)** The proportion of patients with CVC according to the different tumor entities. **(B)** The distribution of port and Hickman access systems among the different patient groups with CVC. **(C)** The distribution of port and Hickman access within the patient subpopulation with thrombosis. **(D)** The proportion of prophylactic anticoagulation at the time of thrombosis according to the different tumor entities. **(E)** The distribution of the different malignancies with regard to the whole cohort (column 1), the whole patient group with central venous access (column 2), and the patient group with central venous access and CVC-associated thrombosis (column 3). **(F)** The proportion of patients with thrombosis according to the different age subgroups.

Systematic searching of all 269 patients with newly diagnosed malignancy and CVC identified 55 patients with thrombosis. Of these 55 patients, 43 had central venous access-related thrombosis, with some patients experiencing CVC-associated thrombosis twice or more, resulting in a total of 52 events in the 43 patients. Table 1 and Figure 2 present the clinical characteristics and distributions of type of disease, CVC-associated thrombosis, and type of CVC.

**Table 1:** **Distribution of CVC associated thrombosis**

	Diagnosis of malignant disease	Patients with CVC	Number of CVC associated thrombosis	Number of patients with CVC associated thrombosis	% of CVC associated thrombosis within the different tumor entities	% of patients with CVC associated thrombosis within the different tumor entities	% of CVC associated thrombosis in all patients with CVC
Lymphoma	35	35 (100%)	7	6	20	17	13%
Leukemia	81	79 (98%)	20	17	25	22	38%
Solid Tumor	116	72 (62%)	5	3	7	4	10%
CNS Tumor	168	68 (40%)	14	12	20	18	27%
Others	48	15 (31%)	6	5	40	33	12%
Total	448	269	52	43			100%

*Abbreviations:* *CNS* central nervous system, *CVC* central venous catheter.

### First episode of CVC-related thrombosis

The group of 43 patients with CVC-associated thrombosis was further analyzed. They included 24 males and 19 females, with a mean age of 9.39 ± 0.95 years (range: 6 months to 18 years). The median time from CVC insertion until catheter-associated thrombosis was 202 ± 32 days, and the median time since diagnosis until catheter-associated thrombosis was 344 ± 53 days. The median CVC follow-up duration was 603 ± 447 days. Twelve patients underwent the CVC insertion operation twice and three patients underwent the operation three times. There was no dependency to the tumor entity. Patients with CVC-associated thrombosis had a mean of 1.6 ± 0.9 catheters inserted. Female patients were significantly older than male patients at the time of thrombosis (11.3 ± 1.4 vs. 7.2 ± 1.2; *p* < 0.05), and the duration from catheter implantation until thrombosis was significant longer in females than males (501 ± 73 vs. 215 ± 66 days; *p* < 0.01). The number of implanted catheters and the thrombosis recurrence rates did not differ with regard to gender or age. Important to note, we did not observed a clinical apparent post-thrombotic syndrome in our patients after CVC-associated thrombosis.

The catheter access veins used in the 52 CVC implantations with CVC-associated thrombosis were as follows: 17 vena cephalica, 11 vena jugularis externa, 11 vena subclavian, 9 vena jugularis interna, 2 vena cava superior, 1 through the vena cava inferior, and 1 vena femoralis. 17 catheters were placed on the left side, and 35 on the right side.

CVC-associated thrombosis occurred in 17 patients with leukemia (n = 20), 6 with lymphoma (n = 7), 3 with solid tumors (n = 5), 12 with central nervous system (CNS) tumors (n = 14), and 5 with other hematologic-oncologic diagnoses, including severe aplastic anemia (SAA), myelo-dysplastic syndrome (MDS), and hemophagocytic lymphohistiocytosis (HLH) (Figure 1). The different tumor entities were not associated with significant differences regarding clinical characteristics (age, thrombophilia, type of catheter, or catheter infections) or treatments performed within the 20 days preceding the appearance of thrombosis (antibiotics, corticosteroids, and chemotherapy in general or surgical approaches), except for the use of asparaginases in patients with leukemia. Even if it failed to reach statistical significance it is mentionable that in our study patients with solid tumors had a lower incidence rate of CVC-associated thrombosis. This may have implications for prophylactic treatment and has to be reevaluated in a greater cohort in future.

Eight of the 17 leukemia patients with CVC-associated thrombosis had received asparaginase within the 20 preceding days. Asparaginase is known to promote thrombophilia by interfering with the coagulation system. Thus, as a preventive measure, each patient undergoing asparaginase treatment had received prophylactic anticoagulation treatment until their coagulation pattern normalized. Despite these measures, 40% (8/20) of the catheter-associated thrombosis in the leukemic patients developed thrombosis in the temporal context of asparaginase medication.

The different tumor entities significantly differed with regard to the use of prophylactic anticoagulation with heparin derivate at the time of thrombosis. Prophylactic anticoagulation was used in 43% of lymphoma patients, 35% of leukemia patients, 28% of CNS tumor patients, and 0% of solid tumor patients (Figure 2D; *P* < 0.05 for between all tumor entities) at the time of thrombosis development. In each case, therapeutic anticoagulation with heparin derivate was administered for a median of 9 days (range: 7 to 14 days).

**Figure 3:**
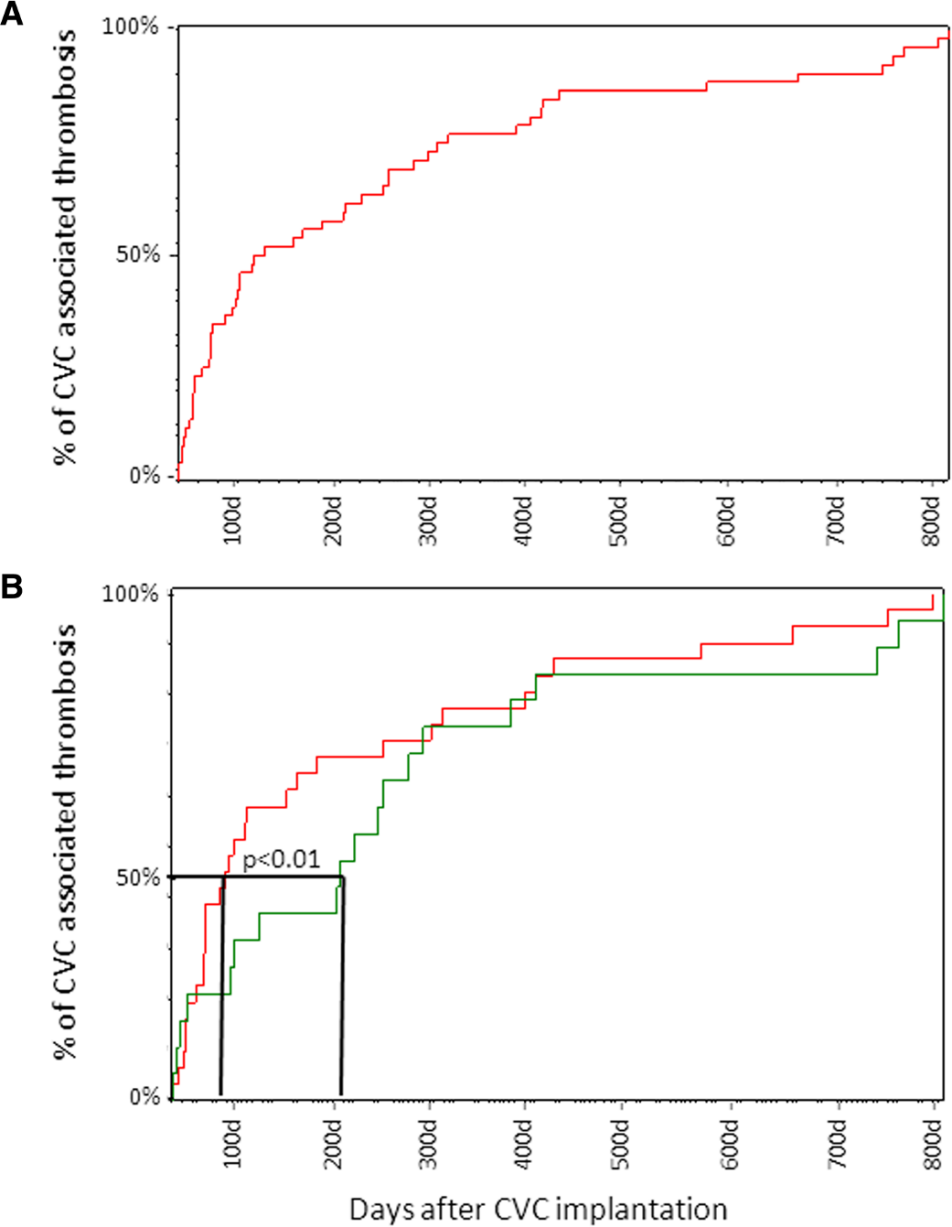
**Days from CVC implantation until appearance of CVC-associated thrombosis. (A)** The days on which thrombosis occurred for each patient. **(B)** The days on which thrombosis occurred in patients with port (red) and Hickman (green) catheters. Analysis of the complete follow-up data revealed no significant differences. Analysis of the time-point at which 50% of thrombosis occurred, revealed that port systems reached a peak of thrombosis events earlier than that with Hickman catheters (*P* < 0.01).

Due to the high inter-individual variety, we could not identify a time-point of increased risk after implantation. Port systems showed an earlier peak of thrombosis occurrence than Hickman catheters; however, the overall prevalence of CVC-associated thrombosis was comparable (Figure 3). Nearly 50% of CVC-associated thrombosis events occurred in ports within the first 100 days after insertion, while Hickman-associated thrombosis on average occurred substantially later at about 230 days after implantation (Figure 3B).

We also found that the time from CVC implantation until thrombosis differed between the different tumor entities. Analyzing the time from diagnosis (in total: 344 ± 53 days) or the time from implantation of central venous access until thrombosis (in total 202 ± 126 days), we observed earlier thrombotic complications in patients with hematological diseases (leukemia: 199 ± 41 days; lymphoma: 125 ± 64 days) and solid tumors (137 ± 80 days) compared to patients with brain tumors (253 ± 61 days). However, due to the small patient numbers, the detected differences failed to reach statistical significance and in addition scatter range was relatively big. However, it seems to be an interesting issue to study as a potential marker of risk and should be studied in an adequate cohort.

### Recurrent CVC-related thrombosis

After thrombosis, 75% of patients received prophylactic anticoagulation if platelet count exhibited above 40/nL. Among these patients, 16% developed a re-thrombosis despite prophylactic anticoagulation. In the lymphoma group, 42% of patients with thrombosis required catheter removal because of re-thrombosis or dysfunction, whereas only 10 to 20% of the central accesses had to be removed in the other groups. We did not find other risk factors for re-thrombosis regarding clinical characteristics in our group. However, it may be limited by the small subgroup and should be re-evaluated in a bigger cohort.

### Comparison between patients with and without CVC-associated thrombosis

When comparing data within the whole patient cohort, which received central venous access (n = 269), the vena subclavia catheters (45/269) showed the highest incidence of thrombosis (11 patients with 45 implanted catheters; 24%), followed by vena jugularis externa and vena cephalica catheters (17 thrombosis/128 implanted catheters; 13%). No differences were found between right- and left-sided catheters.

Furthermore, we did not find significant differences with regard to gender, age, exposure time of CVC, or treatment with high-dose steroids or asparaginase when comparing the CVC-associated thrombosis group with the group of patients with CVC and no CVC-associated thrombosis (data not shown). Regarding the prophylactic heparin use in both groups we did not find a significant differences (68/269 = 25.3% vs. 14/52 = 26.9%). The duration of prophylactic heparin therapy was dependent from co-medication, therapy and the individual risk profile.

Among patients with CVC-associated thrombosis, prothrombin time (89 ± 2.4%), aPTT (34.8 ± 2.5 s), d-dimers (1.6 ± 0.1 mg/L), fibrinogen (2.95 ± 0.2 mg/L), antithrombin III (91.7 ± 3.65%), and platelet count (246000 ± 21000/μL) were within the expected ranges (immediately before catheter insertion and at the time of thrombosis (shown data)) and did not differ significantly from those patients without CVC-associated thrombosis at comparable time points.

Not surprisingly, the surviving patients with progressive disease exhibited significantly more catheter-associated thrombotic events than the patients in complete remission (*p* < 0.05). This observation was even pronounced in patients who died from disease: they had a higher incidence of catheter-associated thrombosis than patients with stable disease and patients in complete remission (*p* < 0.01).

## Discussion

Central venous catheters (CVC) are a well-established and important component of care for pediatric hematology–oncology patients. However, complications are common and must be monitored carefully in order to prevent severe side effects. In this context, one major concern is the prevention of catheter-associated thrombosis. Although the prevalence of deep venous thrombosis and/or thrombotic line occlusion has not yet been determined, these events may be more frequent in children with malignancy and CVC [13, 14] than in a healthy pediatric cohort. Solid data are lacking with regard to the incidence and efficient prevention of catheter-associated thrombosis in the pediatric population. In the review of existing data CVC-associated thrombosis are reported in a big range between 3% up to 40% [15–19]. However, study settings and results are not uniform and valuable data for incidence and risk factors are lacking in the pediatric cohort. Especially pediatric cancer patients seem to have an increased risk for CVC-associated thrombosis compared with non-cancer patients, who received a central venous access [17]. Therefore, it is of special interest in this patient cohort to define risk factors to be able to prevent effectively thrombosis development.

There remains some controversy regarding the best supportive care and treatment plans for pediatric cancer patients with CVC. Thus, an individual investigator may have to rely on personal experience and individual risk–benefit assessment when deciding about prophylactic and therapeutic anticoagulation.

Clinical manifestations due to venous thrombi may be less frequent than actual events. A number of studies have assessed the incidence of VTE associated with long-term CVC in adult cancer patients, with values ranging from 0.3 to 20% [20, 21]. In a study of 72 consecutive autopsy cases, Raad *et al*. [22] reported that 38% of catheterized veins exhibited a fibrin layer on the external surface of all intravascular catheter segments and a mural thrombosis, compared to only 1.4% of controlateral veins. Thus, it appears that these fibrin sleeve thromboses are likely a universal and clinically silent phenomenon. It also seems possible that if they are left untreated, and they persist and grow, they could play an important role in the pathogenesis of thrombosis, infection, and patient outcome. Indeed, several authors have proposed a relationship between catheter-related thrombogenesis and infection [23], and infections in immunosuppressed patients are a proven risk factor for mortality [24, 25].

In pediatric patients, especially in children below three years of age, venous access is often complicated by small vein diameter, particularly in cases of re-implantation. Therefore, it is important to identify VTE risk factors, and to understand how to best prevent catheter-associated VTE. Authors have proposed a number of possible risk factors for CVC-related VTE development, including CVC biocompatibility, number of lumina, catheter tip position, insertion site, CVC insertion techniques, previous CVC insertions and catheter-related complications (mainly CVC malfunction or infections), and high platelet count [21]. Throughout our study period, catheter care was standardized and was performed by the same surgeons, oncologists, and nurses. The tip of a long-term CVC was always located at the junction of the superior caval vein and the right atrium, as correct positioning of the distal catheter tip is associated with a significantly lower rate of CVC-related thrombosis [21, 26].

As reported in previous studies, in general subclavian and jugular access is associated with a lower CVC-related thrombosis incidence than a femoral or brachial access. However, we confirmed that jugular and cephalica access has a lower incidence of CVC-related thrombosis than subclavian [21, 27, 28]. There is some controversy regarding the optimal side for catheter insertion, with some authors reporting that left-sided insertion correlates with an increased risk of thrombotic complications [2]. We did not detect this association in our small pediatric cohort; however, most of the patients had a right-sided catheter, and venous access varied greatly. As in other reports, we found no significant difference in the obstruction-free duration of catheter use between correctly positioned port- and Hickman-catheters [4, 29]. Interestingly, the time of CVC-associated thrombosis occurrence differed significantly between port and Hickman catheters. Nearly 50% of CVC-associated thrombosis events with ports occurred within the first 100 days after insertion, whereas Hickman-associated thrombosis events occurred significantly later at an average of about 230 days after implantation (Figure 3B). We suppose that the earlier peak may be due to the different intensity of therapy—especially among hematologic patients with a port and therapy- and disease-related alterations of coagulation.

Among adult patients, specific malignancies such as ovarian carcinoma or myeloma have been associated with higher incidences of thromboembolia and catheter-associated thrombosis. In the present retrospective analysis we did not identify any patient group as having a higher incidence of CVC-associated thrombosis. However, the pathogenesis of CVC-associated thrombosis is probably multifactorial, with thrombogenesis impacted by CVC-associated features (such as tip position and side of insertion) as well as patient features (such as platelet count, cancer-related and therapy-related coagulation cascade activation, and thrombophilic molecular abnormalities) [30]. However, we did find that patients who underwent a more intensive chemotherapy—such as patients with hematologic malignancies or solid tumors—presented with an earlier VTE occurrence compared to patients with CNS tumors (166 ± 60 vs. 253 ± 61 days after implantation). This fact may relate to the contribution of cancer-related and therapy-related abnormalities of the coagulation cascade to the occurrence of CVC-associated VTE.

Since central venous access is necessary and we cannot influence patient- and cancer-associated features, it is important to discuss other potential options for reducing the incidence of CVC-associated VTE. Hemostasis can be influenced and improved by heparin, vitamin K antagonists, and new generation anticoagulation drugs. In cases without blood regurgitation, mechanical problems must be ruled out (e.g., needle and catheter tip position, and catheter breakage or leakage). Treatment of catheter-associated thrombosis with low-dose fibrinolytic agents, such as streptokinase or urokinase, or with recombinant tissue-type plasminogen activator can be attempted, as it is known to be highly effective and safe in resolving the catheter-related thrombus. Restoration of catheter patency is possible with these agents in two-thirds of cases [4, 23, 31]. This procedure may prevent re-implantation, but will not prevent further growth of the fibrin layer on the external surface of the intravascular catheter. Additionally, a catheter occlusion does not exclude (and may even elevate) the risk of further VTE, and they are often asymptomatic, especially in cancer patients [32, 33]. On the other hand, at the moment clinical data could not show certain evidence, which justified a prophylactic anti-coagulative treatment to prevent silent and asymptomatic VTE, even in patients with catheter obstruction.

CVC-related thrombosis is an important cause of morbidity in cancer patients [20, 34], and therefore many investigators have attempted to identify an effective prophylaxis regimen [20, 23]. The ongoing Thrombotect Study within the ALL-BFM study group is working to address this important issue (J. Greiner, personal communication). Surprisingly, the previously published literature does not support the use of routine thromboprophylaxis for CVC in cancer patients. In our cohort, thromboprophylaxis was performed in patients who were considered to be at a high risk of VTE based on their genetic risk profile, tumor site, or use of asparaginase medication. However, the existing literature contains no evidence supporting the routine use of low-molecular-weight heparin (LMWH), low-dose or adjusted-dose warfarin, continuous unfractionated heparin (UFH), or fibrinolytics to prevent symptomatic CVC-thrombosis [20].

One exception may be the treatment with asparaginase used in leukemia and lymphoma treatment protocols. Up to 25% of all thrombotic events are reported after L-asparaginase infusion and during treatment with corticosteroids [35]. Among the leukemia patients with CVC-associated VTE, 40% developed thrombosis within the temporal context of asparaginase medication. It is well known that L-asparaginase may lead to an increased risk of thrombosis [5], and this knowledge supports the necessity of carefully monitoring patients with CVC who are undergoing L-asparaginase and steroid treatment [36]. Several authors have suggested the use of a prophylactic anticoagulation (as used in the present subjects) or replacement therapy with antithrombin concentrates [37]. Evidence-based guidelines for the prevention and treatment for asparaginase-related VTE are lacking, and randomized trials are urgently needed [38].

Another clinical situation in which prophylactic anticoagulation seems to be justified is in patients who have already had a CVC-related VTE. Without prophylactic anticoagulation, children with CVC-related VTE frequently experience recurrent catheter complications, including re-thrombosis (up to 60%), deep vein thrombosis (DVT; up to 10%), and catheter-associated infections (30%) [7, 39]. The use of prophylactic anticoagulation after CVC-associated thrombosis or VTE (LMWH or oral anticoagulants) seems to decrease the rate of re-thrombosis [20, 21] and this subject should be discussed and further investigated [35, 38, 39].

However, the presented study has several limitations as it is a retrospective study and data were only obtained by chart review. Therefore only symptomatic thromboses were diagnosed. Even if we analyzed a time frame of 5 years the number of CVC-associated thromboses was limited and further diminished by the different types of malignancies as well as by the different prophylactic and therapeutic managements as protocols specifying when to use or not to use antithrombotic prophylaxis are lacking. Statistical analysis can therefore only provide trends, which should be confirmed in a greater prospective cohort with uniform protocols and management regarding antithrombotic therapy and prophylaxis.

## Conclusion

In summary, data about CVC-associated thrombosis and prophylaxis is scarce, especially for pediatric patients. Evidence from adult patients suggests that routine thromboprophylaxis is not justified, especially with regard to the side effects in young hyperactive children. Previous studies emphasize an appropriate catheter position as the most effective measure to prevent CVC-associated thrombosis. Beside the vein position, we were not able to define a risk factor in our oncologic cohort. This may be due to the multimodal pathophysiologic mechanisms, which will lead to thrombophilia in those patients. Pediatric surgeons and oncologists should pay attention to optimal CVC placement and to early symptoms of CVC-related thrombosis in order to avoid relevant CVC-related complications.
